# WISDOM OVER TEA: USE OF COMMUNITY-BASED APPROACHES WITH RURAL ALASKA NATIVE ELDERS

**DOI:** 10.1093/geroni/igae098.1709

**Published:** 2024-12-31

**Authors:** Jordan Lewis, Steffi Kim, Lena Thompson

**Affiliations:** University of Alaska Fairbanks, College of Indigenous Studies, Fairbanks, Alaska, United States; University of Alaska, Anchorage, Alaska, United States; University of Minnesota Duluth, Duluth, Minnesota, United States

## Abstract

Native Elders are often underrepresented in data collection efforts due to distrust of Western research institutions and Western methodologies. Through over 16 years and 162 interviews with Alaska Native (AN) Elders, the Alaska Native Successful Aging Team, led by Dr. Jordan P. Lewis, has been employing strategies to decolonize research. In this presentation, we will share stories and strategies for connecting with rural and remote AN Elders, including the role of sharing (food, tea, gifts, laughter), taking time to build relationships, and the importance of laughter with Elders. Furthermore, we will explain the role of an Elder-led advisory committee and its role in the implementation of Alaska Native driven values-oriented research. The use of community-based approaches, such as talk story, community member involvement, building trust, and maintaining sustainable relationships are necessary steps to conduct research that is Indigenous-driven and beneficial to the communities. Throughout this presentation, we will discuss Indigenous-driven research approaches and the necessity for the increased recognition of research strategies that involve historical, social, cultural, and inter-relational perspectives. Decolonizing approaches to data collection offer space for relationality, and offer a trusting and mutually beneficial environment to conduct research with AN Elders.

